# Letter from Paris

**Published:** 1865-11

**Authors:** 


PARIS, July 15th, 1865.

DEAR DOCTOR:—Since my last letter, Europe has been startled by the appearance of the cholera in Egypt and along the shores of the Mediteranean Sea. It has broken out with great force at Constantinople, and seems to be spreading. In this city, and in London, there are many cases of cholerine, and in the Valle de Grace a case of Asiatic cholera has been reported. Other cases are said to have occurred in the city.

In former visitations, the cholera has progressed regularly from India, along the usual routes of travel. The present epidemic seems to be exceptional in this respect. Its first appearance was among the pilgrims returning from Mecca. Sudden deaths were observed among those who first made their appearance at Cairo and Alexandria, but the first recorded cases occurred, at the latter place on the 10th of May, and at the former on the 26th of the same month.

It is well known, that every faithful Mussulman considers it a sacred duty to make, at least once in his lifetime, a pilgrimage to the holy city, the accomplishment of which gives him a special claim to sanctity. During the closing portion of this pilgrimage, he imposes upon himself the severest privations; walks barefooted and bareheaded, with shaven crown, and with no protection, beneath the burning rays of a tropical sun; religiously abstains from destroying the prolific vermin engendered amid the filth of the motley crowd of Asiatics, Africans, and Europeans, and restricts his diet to the poorest and coarsest fare. From 150,000 to 300,000, after these long fatigues, fastings and exposures, crowded into the smallest possible space, and wrought up to the highest pitch of excitement, join in the religious ceremonies which terminate in the sacrifice of as many sheep as there are worshippers. They may neither eat nor sell the offering, and the vast heap of slaughtered animals, with the thousands of human bodies, fester in the hot sun and breed pestilence and death, bringing with it the martyr's crown to the weary followers of the Prophet.

It is not strange that epidemics should be developed under such circumstances, and especially this year, for I learn that the usual appropriation of $60,000 to the Governor of Mecca, for the purpose of improving the sanitary condition of the city, has been withheld. The disease continued to increase in Cairo and Alexandria until the waters of the Nile began to rise, and, at last accounts, the mortality was diminishing.

During the last year, the cholera has been more than usually prevalent in India, but has not extended westward, nor has the disease developed among the pilgrims, so far as I can learn, extended eastward. In fact, so far, it seems to be confined to the basin of the Mediteranean Sea. I think it likely that Northern and Western Europe will escape this year, and it is possible, though I think it not probable, that the coming winter may destroy or render inert the producing causes of the pestilence.

Of one thing, however, I am quite certain: Europe is ripe for the development of epidemics. In Russia, during the Spring and early Summer, a disease of a typhoid character has been prevalent. This has been called the Russian plague, but is only a modified form of the fevers of Liverpool and Naples, mentioned in my previous letters. In London and Paris, the same class of diseases are now epidemic; while in Prussia and Central Europe, cerebro-spinal meningitis has prevailed to an alarming extent. There is, therefore, a general tendency to epidemics. The vitality of the great mass of the people seems to be lowered; diarrhœa and dysentery have been more than usually common in all the large cities, and the percentage of mortality from these diseases much above the average. There is, therefore, I think, reason to fear that Northern and Western Europe will suffer from the cholera during the coming year. In fact, the disease already exists in Paris and London. Strict quarantine measures have been instituted, but they have not prevented the fiend from leaping, at one bound, from Ancona to the banks of the Seine, not, it is true, as an epidemic, but presenting, rather, one of those cases of spontaneous generation that have so recently puzzled the French academicians. I have no doubt but that a judicious quarantine is useful, but if, as we have seen, the pestilence has been developed *de novo* amid the filth and wretchedness of 150,000 fanatics on the borders of the Red Sea, I know of no reason why it may not also be produced among the lazzaroni of Naples, or in the dark, dirty, crowded lanes of London and Edinburgh.

The commission charged with the verification of deaths in

Paris, has recently presented to the prefect of the Seine, a memoir on the mortality of the French capital during the twenty-four years ending with 1863. In the beginning of the eighteenth century, the mortality was 1 in 28; in 1840, 1 in 36; and in 1863, 1 in 40. The causes that have effected this improvement are various, but may be traced directly, or indirectly, to the progress of medical science and practice. Public hygiene is taught as an important department in *l'ecole de medicine*, and hygienic laws are promulgated among the people, while the authorities, to a greater extent than formerly, look after the necessities and comforts of the lower classes. Paris, itself, during the last few years, has undergone a great physical change; many of the old, narrow, crooked streets are replaced by wide, airy boulevards; parks and gardens, to the extent of 1-24th part of the whole city, have been laid out and planted with trees; the sewerage has been largely extended; an abundance of pure water supplied; the Seine, itself, kept free from the filth of the city; the poorer classes more abundantly supplied with good, wholesome food; the density of the population materially diminished, by extending the area of the city; private dwellings have been made subject to public inspection, and the administration of public charities is thorough and efficient, securing to the poorest child of this great city, every day if need be, the benefit of the best medical and surgical talent of Paris. The *Hotel Dieu* is about being rebuilt at a cost of not less than $4,000,000. *La Charite* is also undergoing regeneration and enlargement.

I have been more particularly interested in the wards of Grisolle, Trouseau, Moneret, and of Piory at the *Hotel Dieu*, and of Bouchet, at *l'Hopital des Enfants Malades*, but have seen most of the distinguished physicians and surgeons of the city. Velpeau is still active, visiting his wards at *La Charite*, and giving clinical instruction every morning. I heard him lecture for an hour to an audience of six persons. His eyesight is still good, and his hand steady, though he operates very slowly. Jobert (de Lamballe), at the *Hotel Dieu*, scolds and frets, as I presume he always has done, abuses his internes, and makes himself generally disagreeable. I saw him inject several cases of hydrocele, and one lumbar abscess, with a solution of ammonia. He stated that the inflammation following the injection of ammonia was more plastic than that produced by tr. iodine. I cannot learn that other surgeons here share his opinion. For hydrocele, MAISONEUVE and DESORMEAUX make an application of solid nit. silver, through a canula, to the internal walls of the sac.

MAISONEUVE gave me the statistics of his operations for the cure of varicose veins by injections of per-chloride of iron. He reports 365 operations, 364 cures, and one death. In the fatal case, the tr. iodine was used, by mistake, for the per-chloride of iron. A surgeon of large experience, who has had good opportunities to observe his cases, expresses some doubt as to all the others being *cures*. The operation, if carefully conducted, seems to be safe and is probably as successful as any other.

I have had frequent opportunities of seeing DESORMEAUX use his *endoscope*. He demonstrates the inner walls of the bladder and the mucous membrane of the urethra, throughout its whole extent. I also saw him operate through the instrument for stricture. In chronic inflammations of the prostatic portion, usually so troublesome, the *endoscope* frequently reveals granulations similar to those of granulated eyelids. In such cases, he applies, through the tube, the sulph. of copper in substance.

The Medical Faculty are, as a general rule, opposed to specialties. They say that specialists have never yet established any important principle in pathology or therapeutics, though they may attain great dexterity in certain manipulations. As an illustration of this latter proposition, I may mention the younger DESMARRES. His operations on the eye are certainly wonderful. I have been especially interested in the clinic of FAUVEL, for diseases of the throat and larynx. He is very expert in the use of the laryngoscope, and gave me an opportunity to see some cases of great interest. Among others, a polypus, situated upon one of the vocal chords, and producing complete aphonia. The removal of the morbid growth was followed by restoration of the voice.

In the Academy of Medicine, at the last meeting, the subject of discussion was thoracentesis, an operation quite frequently performed both in the hospitals and private practice, in this city. M. GUERIN presented a new instrument, intended to prevent the introduction of air into the pleural sac, and also to evacuate, slowly, the contained fluid. At previous meetings of the Academy, the subject of *aphasia* has excited a very general interest. This term has been applied, by TROUSSEAU, to a loss of the power of speech, without lesion of the organs of voice. In one of the wards at the *Hotel Dieu*, I saw a man who could only speak the words "*tout le meme*," on coming in. He was subsequently able to add one or two words, and will now, occasionally, speak a whole sentence, always commencing with the word *tout*. He understands what is said, and seems greatly depressed, sometimes bursting into tears, after fruitless efforts to answer. J.

